# Response of soil microbial community to application of biochar in cotton soils with different continuous cropping years

**DOI:** 10.1038/s41598-017-10427-6

**Published:** 2017-08-31

**Authors:** Guangming Han, Jiayang Lan, Quanqiu Chen, Cui Yu, Shu Bie

**Affiliations:** Industrial Crops Institute of Hubei Academy of Agricultural Sciences/Key laboratory of Cotton Biology and Breeding in the Middle Reaches of the Changjiang River, Wuhan, 430064 China

## Abstract

The bacterial community in soils of cotton that have continuously been cropped for 2 years, 6 years, 11 years and 14 years and treated with biochar (B0, 0 t·ha^−1^; B1, 12.5 t·ha^−1^; and B2, 20 t·ha^−1^) was investigated using next-generation sequencing. Of the 45 bacterial genera (relative abundance ratio of genera greater than 0.3%), 21 genera were affected (p < 0.05) by the biochar treatment, whereas 20 genera were affected by the continuous cropping. Between the soils that have been continuously cropped for 2 years and 14 years, 12 different genera were significantly observed (p < 0.05), and 6 genera belonged to the phylum *Acidobacteria*. The relative abundance of *Sphingomonas* and *Pseudomonas* in the biochar-treated soils was significantly higher than that in the soil without biochar treatment (p < 0.05), and the relative abundance of *Sphingomonas* and *Pseudomonas* in soils that have been continuously cropped for 2 years and 6 years was significantly higher than that in the soils continuously cropped for 11 years and 14 years (p < 0.05). The results suggest that the biochar application has a significant impact on the soil bacterial community, which may improve the microbial diversity of continuous cropping systems in cotton soils.

## Introduction

Biochar has been recommended as a soil modification additive in several soil management regimes^1, 2^, and biochar application has achieved a great deal of positive effects, including improvements in the soil fertility, increases in the size of the soil microbial community^3–5^, and decreases in nitrous oxide (N_2_O) and methane (CH_4_) emissions^1, 6, 7^. In particular, modification of soils with biochar has been shown to increase plant yield and modify the soil habitat of microbes, protecting microbes from predation by soil microarthropods^8, 9^, thus altering the soil microbial activity and community structure^10, 11^. Lehmann *et al*.^12^ reported that application of biochar increases microbial activity and biomass and changes the microbial community composition and abundance^11^. However, the beneficial effect of biochar on the soil environment depends on the type of biochar, application rate, soil type and plant response^3^. More recently, compounds inhibiting microbial activity have been found in the biochar^13, 14^ as well as in the soil (released after biochar introduction)^15^. Regardless, these changes will likely spread unequally across different phylotypes or functional groups. In addition, little is known about how specific microorganisms are affected by such changes^8^.

Hubei Province is the second-largest production area of cotton in China. The practice of continuous cotton cropping is significant in this area. Continuous cropping has resulted in a decline in cotton production, in quality and in soil microbial diversity. At the same time, the diseases and insect pests of cotton, such as Verticillium wilt and Fusarium wilt, commonly occur and affect the income of farmers. Continuous cropping has been one of the key issues in need of solving in cotton production industry. Recently, organic alterations have provided a substrate for the burgeoning soil microbial community, resulting in the development of a self-sustaining, belowground microbial community that will generally increase the success of reclamation efforts^16^. Biochar, as a new type of environmental friendly soil-improvement material, is widely used in agricultural production, especially for improving soil quality. For decades, research has focused on the physical and chemical soil variables, with less attention paid to those effects on microorganisms. In the present study, we determined the relative effects of biochar application on the composition of the soil microbial community in different cotton soils that have been continuously cropped system in Hubei Province. This study aimed to characterize the soil bacterial communities among the different continuous cotton soil field cropping systems treated with biochar to answer the following questions: (i) Does the overall soil bacterial community differ among different continuous cotton field systems? (ii) Can biochar treatments change the soil bacterial community of different continuous cotton field systems? This information will improve our understanding of the relationship between biochar application and soil microbial ecology in continuous crop systems of cotton.

## Results

### Sequence data and bacterial taxonomic richness

**Figure 1:**
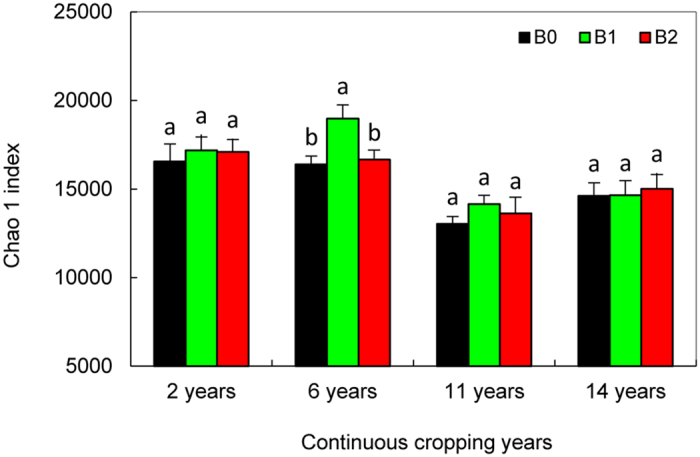
The Chao1 index in cotton soils that have been continuously cropped for 2 years, 6 years, 11 years and 14 years and treated with biochar (B0, 0 t·ha^−1^; B1, 12.5 t·ha^−1^; and B2, 20 t·ha^−1^).

A total of 1,669,315 paired-end 250 bp reads were acquired, and the average read length per sample was 0.16 Gb, with 87,323 to 204,627 raw reads in cotton soils with different continuous cropping years and biochar treatments (Table S1). After the initial quality control process, 1,608,450 high-quality sequences were obtained. On average, 134,038 sequences were obtained per sample. Based on 97% species similarity, 6552 to 9420 operational taxonomic units (OTUs) were separately obtained from the samples of different continuously cropped cotton soils with biochar treatment (Table S1). The average length of the sequence reads was 440 bp, and they were classified into different taxonomic groups using Uclust^17^. The bacterial diversity is reflected by the Chao1 index; the Chao1 index in the soils cropped continuously for 2 years and 6 years was higher than that in the soils continuously cropped for 11 years and 14 years (Fig. 1), with significant differences (except for the 6-B0 soil). This finding indicates that the bacterial diversity decreased during the continuous years.

### Effect of biochar on the bacterial community composition

**Figure 2:**
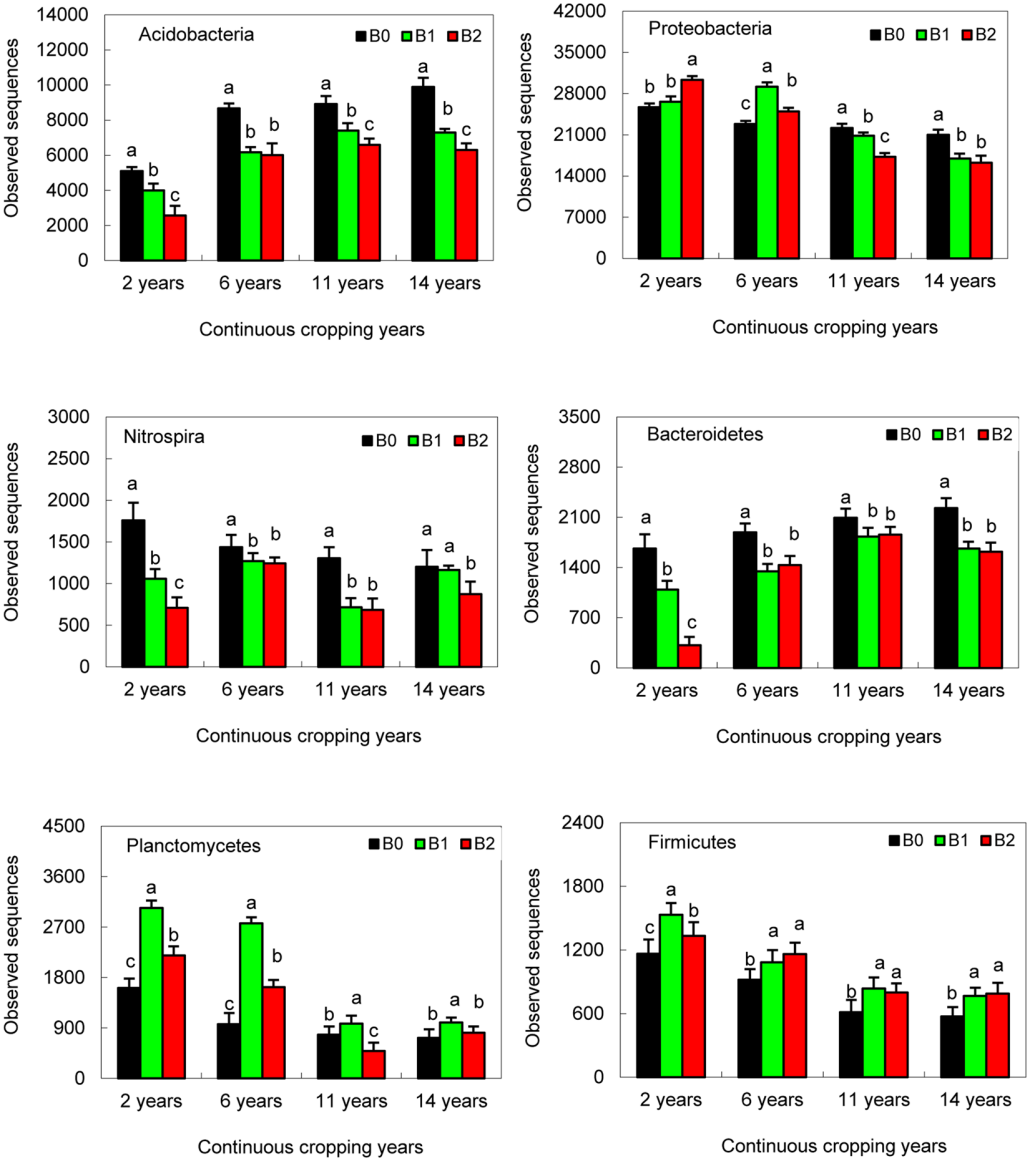
The abundance of *Acidobacteria*, *Proteobacteria*, *Nitrospira*, *Bacteroidetes*, *Planctomycetes* and *Firmicutes* in cotton soils that have been continuously cropped for 2 years, 6 years, 11 years and 14 years and treated with biochar (B0, 0 t·ha^−1^; B1, 12.5 t·ha^−1^; and B2, 20 t·ha^−1^).

Results shown in Fig. S1 describe the distribution of the DNA sequences into phyla. A total of 34 phyla were shared by the 12 soil samples. The main phyla were as follows: *Proteobacteria*, *Acidobacteria*, *Actinobacteria*, *Nitrospira*, *Bacteroidetes*, *Gemmatimonadetes*, *Planctomycetes*, *Firmicutes* and *Verrucomicrobia*. *Proteobacteria* was the most dominant among the 34 phyla (p < 0.05) in the samples and comprised 39% to 54.5% of the total sequences. *Acidobacteria* was the second-largest phylum in all groups, comprising approximately 14.2% to 21.3% of the different groups (Fig. 2). Seven phyla (*Proteobacteria*, *Acidobacteria*, *Nitrospira*, *Bacteroidetes*, *Planctomycetes*, *Firmicutes* and *Verrucomicrobia*) differed (p < 0.05) between the continuous cropping year and biochar treatment (Table S2). The relative abundance of *Proteobacteria*, *Nitrospira*, *Planctomycetes* and *Firmicutes* was significantly reduced by the continuous year, while the relative abundance of *Acidobacteria* and *Bacteroidetes* increased by the continuous year (Fig. 2). Biochar treatment increased the relative abundance of *Proteobacteria* in the soils continuously cropped for 2 years and 6 years but not in the continuous 11-year and 14-year soils. Biochar treatment also increased the relative abundance of *Planctomycetes* and *Firmicutes*, especially for the B1 (12.5 t·ha^−1^) treatment regarding *Planctomycetes*. However, biochar treatment reduced the relative abundance of *Acidobacteria* and *Bacteroidetes* in different continuous-year soils.

At the genus level, a total of 853 genera were identified from all the samples, regardless of the treatments administered. The 46 most abundant genera (with relative abundance of more than 0.3%), comprising more than 66.6% of the total sequences, are shown in Fig. S2. Of the 46 genera, 29 were affected by the continuous cropping year, biochar treatment and interactions between the continuous cropping year and biochar treatment (p < 0.05). Among these genera, 20 were affected by the continuous cropping year, 21 by the biochar treatment and 12 by the interactions between continuous cropping year and biochar treatment (Table 1). The relative abundance of *Gemmatimonas*, *Nitrospira*, *Sphingomonas*, *Pseudomonas*, *Dongia*, *Phaselicystis*, *Kofleria*, *Nitrosospira*, *Geobacter*, *Ramlibacter*, *Novosphingobium* and *Ohtaekwangia* was significantly reduced by the continuous year, while the relative abundance of *Gp6*, *Gp4* and *Gp10* was increased by the continuous year. Biochar treatment could increase the relative abundance of *Gemmatimonas*, *Nitrospira*, *Sphingomonas*, *Pseudomonas* and *Anaeromyxobacter* but reduce the relative abundance of *Rhodoplanes*, *Ilumatobacter*, *Gp6*, *Gp4* and *Gp10* (Table 2).

**Table 1:** ANOVA for genus abundance.

Phyla	Genus	p value (*p < 0.05, **p < 0.01)		
		Continuous cropping years	Biochar treatments	Continuous cropping years *biochar treatments
*Gemmatimonadetes*	*Gemmatimonas*	<0.001**	<0.001**	<0.001**
*Nitrospira*	*Nitrospira*	0.002**	<0.001**	<0.001**
*Proteobacteria*	*Sphingomonas*	<0.001**	<0.001**	0.037*
	*Pseudomonas*	<0.001**	<0.001**	<0.001**
	*Steroidobacter*	<0.001**	0.068	0.057
	*Dongia*	0.042*	0.094	0.135
	*Anaeromyxobacter*	0.054	0.001**	0.051
	*Skermanella*	0.068	0.004**	0.054
	*Lysobacter*	0.009**	0.003**	0.001**
	*Phaselicystis*	0.012*	0.024*	0.078
	*Kofleria*	0.025*	0.088	0.020*
	*Nitrosospira*	0.032*	0.026*	0.023*
	*Rhizobium*	0.053	0.028*	0.051
	*Geobacter*	0.058	0.028*	0.035*
	*Rhodoplanes*	0.064	0.041*	0.056
	*Ilumatobacter*	0.072	0.045*	0.051
	*Ramlibacter*	0.082	0.049*	0.068
	*Novosphingobium*	0.075	0.041*	0.058
*Acidobacteria*	*Gp6*	0.031	0.023*	0.013*
	*Gp4*	0.001**	<0.001**	0.152
	*Gp10*	0.044*	0.084	0.067
	*Gp3*	0.025*	0.068	0.072
	*Gp7*	0.026*	0.027*	0.061
	*Gp16*	0.042*	0.032*	0.029*
	*Gp5*	0.041*	0.052	0.035*
	*Gp17*	0.042*	0.055	0.056
*Firmicutes*	*Pasteuria*	<0.001**	<0.001**	0.164
*Planctomycetes*	*Gemmata*	0.001**	0.134	<0.001**
*Bacteroidetes*	*Ohtaekwangia*	0.029*	0.010*	0.052

**Table 2:** The relative abundance of genera (had significant differences) in cotton soils that have been continuously cropped for 2 years, 6 years, 11 years and 14 years treated with biochar (B0, 0 t·ha^−1^; B1, 12.5 t·ha^−1^; and B2, 20 t·ha^−1^).

Genera	2 years			6 years			11 years			14 years		
	B0	B1	B2	B0	B1	B2	B0	B1	B2	B0	B1	B2
*Gemmatimonas*	1812 ± 23^b^	2056 ± 40^a^	1731 ± 17^c^	1182 ± 24^c^	1712 ± 16^b^	1860 ± 17^a^	1142 ± 20^c^	1163 ± 7^bc^	1314 ± 17^a^	384 ± 13^c^	2009 ± 14^a^	1632 ± 11^b^
*Nitrospira*	2669 ± 91^a^	535 ± 62^c^	1743 ± 66^b^	1503 ± 89^a^	714 ± 97^b^	685 ± 82^b^	1474 ± 11^a^	1162 ± 12^b^	1271 ± 11^b^	2659 ± 56^a^	709 ± 50^c^	1757 ± 82^b^
*Sphingomonas*	3161 ± 92^b^	2884 ± 85^c^	3712 ± 62^a^	1508 ± 91^c^	2150 ± 98^b^	2480 ± 82^a^	849 ± 81^c^	1420 ± 79^b^	1914 ± 62^a^	446 ± 99^c^	2422 ± 61^a^	2167 ± 99^b^
*Pseudomonas*	1385 ± 84^c^	2968 ± 92^a^	1800 ± 72^b^	538 ± 62^c^	2380 ± 94^a^	1981 ± 67^b^	390 ± 86^c^	739 ± 91^b^	1656 ± 48^a^	142 ± 66^c^	891 ± 64^b^	1151 ± 96^a^
*Steroidobacter*	955 ± 21^a^	739 ± 16^b^	636 ± 11^c^	721 ± 11^bc^	693 ± 17^c^	762 ± 20^a^	1129 ± 19^a^	744 ± 21^c^	904 ± 15^b^	1375 ± 12^b^	1445 ± 15^a^	1033 ± 13^c^
*Dongia*	1256 ± 18^a^	871 ± 13^bc^	862 ± 16^c^	804 ± 15^c^	863 ± 15^b^	876 ± 11^ab^	786 ± 20^bc^	770 ± 24^c^	1259 ± 25^a^	253 ± 4^c^	1312 ± 14^a^	1101 ± 14^b^
*Anaeromyxobacter*	1036 ± 17^c^	1733 ± 16^b^	1971 ± 14^a^	596 ± 10^c^	773 ± 7^a^	763 ± 12^ab^	197 ± 6^c^	354 ± 9^a^	305 ± 7^b^	218 ± 5^c^	623 ± 10^ab^	632 ± 8^a^
*Skermanella*	674 ± 9^bc^	688 ± 7^ab^	700 ± 7^a^	1606 ± 12^a^	349 ± 7^c^	504 ± 9^b^	876 ± 9^a^	502 ± 8^c^	535 ± 7^b^	412 ± 8^c^	994 ± 12^a^	793 ± 14^b^
*Lysobacter*	267 ± 8^c^	865 ± 12^a^	382 ± 7^b^	358 ± 7^c^	585 ± 7^b^	601 ± 8^a^	710 ± 7^a^	388 ± 7^c^	456 ± 7^b^	69 ± 3^c^	665 ± 12^a^	484 ± 9^b^
*Phaselicystis*	507 ± 6^a^	444 ± 9^c^	487 ± 7^b^	397 ± 6^c^	454 ± 9^a^	431 ± 5^b^	271 ± 7^c^	375 ± 9^b^	489 ± 10^a^	148 ± 7^c^	632 ± 7^a^	523 ± 8^b^
*Kofleria*	511 ± 7^abc^	502 ± 7^c^	517 ± 4^a^	375 ± 7^bc^	492 ± 8^a^	363 ± 5^c^	330 ± 6^c^	495 ± 8^a^	419 ± 5^b^	166 ± 6^c^	481 ± 9^ab^	487 ± 6^a^
*Nitrosospira*	808 ± 6^a^	514 ± 5^b^	273 ± 6^c^	377 ± 10^c^	604 ± 7^a^	548 ± 14^b^	344 ± 6^b^	278 ± 8^c^	501 ± 9^a^	103 ± 2^c^	422 ± 7^a^	382 ± 7^b^
*Rhizobium*	882 ± 11^a^	155 ± 6^c^	440 ± 6^b^	160 ± 4^bc^	164 ± 4^b^	279 ± 8^a^	251 ± 7^b^	155 ± 4^c^	408 ± 6^a^	147 ± 6^c^	624 ± 6^a^	302 ± 5^b^
*Geobacter*	643 ± 15^b^	155 ± 6^c^	441 ± 6^b^	160 ± 4^bc^	164 ± 4^b^	279 ± 8^a^	251 ± 7^b^	155 ± 4^c^	409 ± 6^a^	147 ± 6^c^	624 ± 6^a^	302 ± 5^b^
*Rhodoplanes*	406 ± 7^a^	110 ± 3^c^	206 ± 4^b^	301 ± 5^a^	183 ± 3^c^	220 ± 6^b^	345 ± 5^a^	282 ± 4^b^	224 ± 5^c^	362 ± 5^a^	231 ± 6^c^	242 ± 9^bc^
*Ilumatobacter*	565 ± 7^a^	198 ± 4^c^	297 ± 5^b^	286 ± 9^a^	130 ± 6^c^	180 ± 6^b^	273 ± 8^a^	185 ± 4^c^	108 ± 6^c^	234 ± 5^a^	165 ± 4^bc^	159 ± 8^c^
*Ramlibacter*	345 ± 6^b^	463 ± 6^a^	115 ± 3^c^	216 ± 12^c^	627 ± 18^a^	236 ± 6^bc^	107 ± 6^c^	217 ± 6^b^	396 ± 7^a^	35 ± 2^c^	281 ± 4^b^	298 ± 7^a^
*Novosphingobium*	509 ± 11^a^	431 ± 10^b^	203 ± 5^c^	188 ± 4^b^	171 ± 4^c^	271 ± 7^a^	180 ± 8^c^	207 ± 6^b^	443 ± 9^a^	80 ± 3^c^	429 ± 13^a^	211 ± 4^b^
*Gp6*	2617 ± 21^a^	2269 ± 24^b^	1052 ± 16^c^	3158 ± 19^a^	2551 ± 28^c^	2698 ± 24^b^	3168 ± 23^a^	2104 ± 21^c^	2783 ± 22^b^	4354 ± 30^a^	3151 ± 34^c^	3494 ± 18^b^
*Gp4*	1309 ± 11^ab^	1369 ± 25^a^	199 ± 5^c^	1428 ± 15^a^	860 ± 13^b^	549 ± 8^c^	1547 ± 18^a^	1003 ± 10^b^	1142 ± 11^c^	1805 ± 17^a^	1021 ± 12^c^	1094 ± 16^b^
*Gp10*	646 ± 11^b^	709 ± 8^a^	409 ± 6^c^	853 ± 7^a^	475 ± 6^c^	845 ± 7^ab^	1121 ± 13^a^	1019 ± 13^b^	914 ± 8^c^	1392 ± 14^a^	939 ± 10^c^	991 ± 13^b^
*Gp3*	991 ± 10^a^	655 ± 7^c^	730 ± 7^b^	642 ± 16^a^	548 ± 9^bc^	539 ± 13^c^	292 ± 6^c^	493 ± 9^b^	660 ± 11^a^	119 ± 7^c^	426 ± 13^a^	382 ± 14^b^
*Gp7*	744 ± 9^a^	538 ± 12^c^	552 ± 21^bc^	280 ± 6^c^	549 ± 18^ab^	475 ± 7^a^	147 ± 6^c^	365 ± 8^b^	398 ± 8^a^	118 ± 4^c^	284 ± 7^a^	237 ± 6^b^
*Gp16*	288 ± 6^b^	307 ± 6^a^	144 ± 5^c^	431 ± 6^a^	231 ± 6^c^	308 ± 5^b^	542 ± 7^a^	422 ± 7^b^	252 ± 7^c^	645 ± 8^a^	479 ± 13^b^	425 ± 6^c^
*Gp5*	427 ± 5^a^	323 ± 5^c^	359 ± 8^b^	357 ± 8^b^	314 ± 5^c^	422 ± 6^a^	379 ± 10^a^	249 ± 7^c^	368 ± 7^ab^	516 ± 7^a^	444 ± 7^b^	344 ± 11^c^
*Gp17*	277 ± 6^b^	206 ± 5^c^	353 ± 8^a^	282 ± 6^a^	207 ± 7^bc^	196 ± 7^c^	617 ± 9^a^	277 ± 8^b^	236 ± 7^c^	181 ± 6^c^	502 ± 8^a^	388 ± 6^b^
*Pasteuria*	783 ± 8^a^	398 ± 6^c^	454 ± 7^b^	400 ± 6^b^	453 ± 7^a^	157 ± 4^c^	506 ± 7^a^	350 ± 8^c^	449 ± 10^b^	1993 ± 16^a^	15.5 ± 15^b^	780 ± 14^c^
*Gemmata*	238 ± 7^c^	338 ± 6^b^	562 ± 8^a^	1000 ± 11^a^	243 ± 6^b^	114 ± 3^c^	692 ± 9^a^	282 ± 7^b^	261 ± 6^c^	72 ± 2^c^	1037 ± 9^a^	522 ± 7^b^
*Ohtaekwangia*	478 ± 8^b^	566 ± 7^a^	333 ± 6^c^	387 ± 7^b^	467 ± 9^a^	296 ± 7^c^	151 ± 5^c^	234 ± 6^b^	643 ± 9^a^	80 ± 2^c^	667 ± 6^a^	510 ± 7^b^

The data are expressed as the means ± SD (n = 3). The superscript letters that differ within a column indicate significant differences between treatments (p < 0.05).

**Figure 3:**
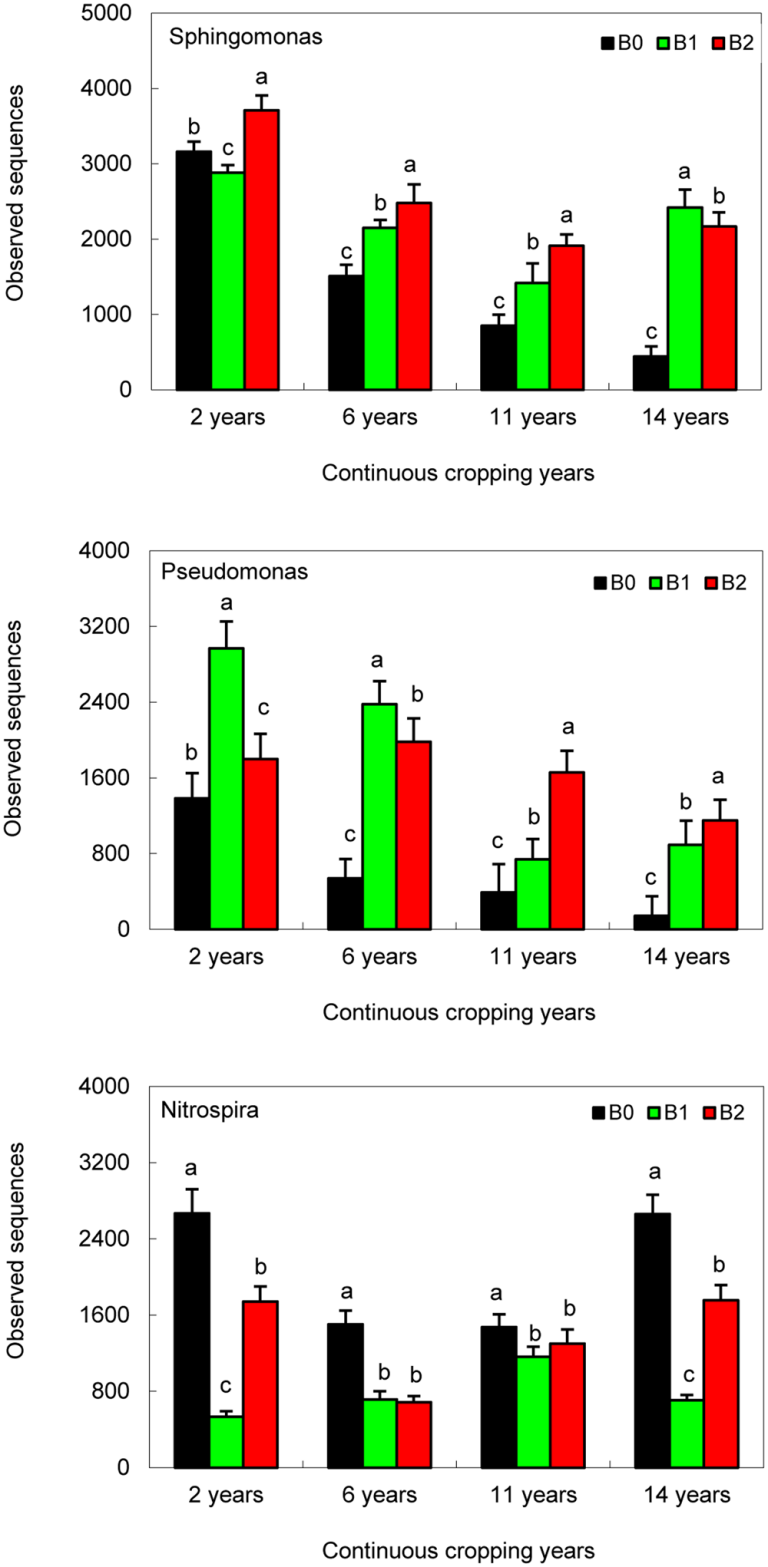
The abundance of *Sphingomonas*, *Pseudomonas* and *Nitrospira* in cotton soils that have been continuously cropped for 2 years, 6 years, 11 years and 14 years and treated with biochar (B0, 0 t·ha^−1^; B1, 12.5 t·ha^−1^; and B2, 20 t·ha^−1^). Error bars indicate standard deviation (SD) (n = 3). Different letters above the bars denote statistical significance at p < 0.05, according to the LSD test.

The five most abundant genera were *Sphingomonas*, *Gemmatimonas*, *Nitrospira*, *Pseudomonas* and *Gp6* (Fig. S2). The relative abundance of *Sphingomonas* and *Pseudomonas* in the biochar-treated soils was significantly higher than that the soils without biochar treatment (Fig. 3, p < 0.05), and the relative abundance in the cotton soils continuously cropped for 2 years and 6 years was significantly higher than that of the 11-year and 14-year continuously cropped soils (p < 0.05). However, the relative abundance of *Nitrospira* in the soils without biochar treatment in different continuous cropping years was significantly higher compared with that of the biochar-treated soils (Fig. 3, p < 0.05).

**Figure 4:**
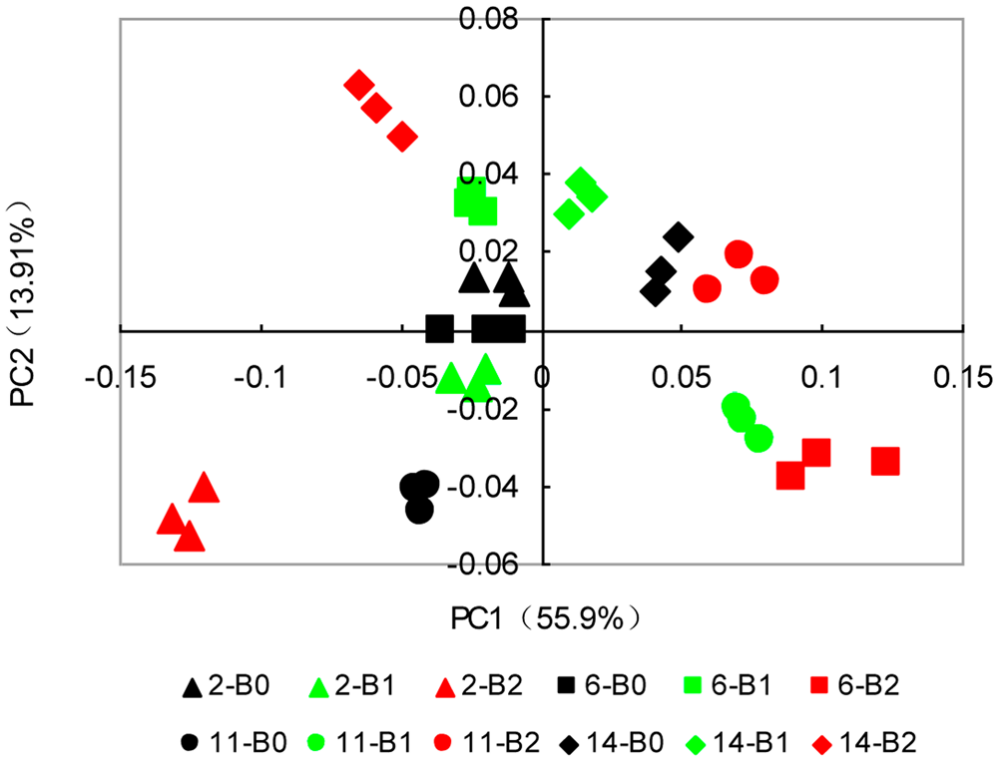
PCoA of Bray-Curtis distances for the bacteria in cotton soils that have been continuously cropped for 2 years, 6 years, 11 years and 14 years and treated with biochar (B0, 0 t·ha^−1^; B1, 12.5 t·ha^−1^; and B2, 20 t·ha^−1^).

Eight genera significantly differed (p < 0.05) between soil continuously cropped for 2 years and 6 years, and 10 genera differed between 2-year and 11-year continuously cropped soils (Table 3). Most of the genera belonged to the phylum *Proteobacteria*. However, between soils continuously cropped for 2 years and 14 years, 12 significantly different genera (p < 0.05) were identified, with 6 of those genera belonging to the phylum *Acidobacteria*. The relative fold changes in *Pseudomonas* between soils continuously cropped for 11 years and 2 years and between 14-year and 2-year soils were −19.81 and −19.02, respectively, and the differences were significant (p < 0.05). The soil samples were separated into categories, which matched their different continuous cropping year (Fig. 4). The separation was clearer for the cotton soils continuously cropped for 11 years and 14 years than for that of 6-year and 2-year soils, suggesting that the number of years of continuous cropping influenced the bacterial community composition.

**Table 3:** Comparison (*t*-tests and Metastats) of different continuous cropping years for genus abundance.

Phyla	Genus	Relative fold change	p value (*p < 0.05, **p < 0.01)
		6 years/2 years	
*Planctomycetes*	*Gemmata*	19.19	0.003**
*Proteobacteria*	*Steroidobacter*	−2.17	0.016*
	*Sphingomonas*	−19.07	0.010**
	*Skermanella*	18.90	0.010**
	*Pseudomonas*	−2.26	0.021*
	*Kofleria*	−1.96	0.012*
*Nitrospira*	*Nitrospira*	−18.61	0.020*
*Firmicutes*	*Pasteuria*	−2.94	0.020*
**Phyla**	**Genus**	**Relative fold change**	p value (*p < 0.05, **p < 0.01)
		**11 years/2 years**	
*Gemmatimonadetes*	*Gemmatimonas*	−2.20	0.003**
*Proteobacteria*	*Pseudomonas*	−19.81	<0.001**
	*Anaeromyxobacter*	−4.57	0.005**
	*Lysobacter*	1.33	0.015*
	*Steroidobacter*	1.04	0.043*
	*Phaselicystis*	−1.99	0.003**
*Planctomycetes*	*Gemmata*	1.46	0.007**
*Nitrospira*	*Nitrospira*	−1.70	0.011*
*Acidobacteria*	*Gp17*	1.35	0.011*
	*Gp6*	10.33	0.031*
**Phyla**	**Genus**	**Relative fold change**	p value (*p < 0.05, **p<0.01)
		**14 years/2 years**	
*Proteobacteria*	*Steroidobacter*	3.58	0.008**
	*Nitrosospira*	−2.75	0.010**
	*Pseudomonas*	−19.02	0.010**
	*Gemmata*	−1.31	0.032*
	*Lysobacter*	1.53	0.034*
*Acidobacteria*	*Gp4*	3.62	0.001**
	*Gp10*	1.74	0.003**
	*Gp3*	−4.20	0.004**
	*Gp7*	−2.81	0.005**
	*Gp16*	1.87	0.012*
	*Gp17*	−1.75	0.012*
*Firmicutes*	*Pasteuria*	3.26	0.007**

### Biochar has a significant effect on soil bacterial composition

Twenty-one genera were affected (p < 0.05) by biochar treatment (Table 1). Specifically, 10 different genera were observed (p < 0.05) between B1- and B0-treated soils, and 13 genera between B2- and B0-treated soils based on T-test results (Table 4). Among the 21 genera, the relative fold changes of *Pseudomonas*, *Sphingomonas*, *Pasteuria* and *Nitrospira* between B2- and B0-treated soils were 12.45, 19.40, −19.48 and −17.75, respectively, and the differences were significant (p < 0.05). Our data suggest that the genera in the biochar-treated soil were significantly different. *Pseudomonas*, a major rhizosphere-promoting bacteria, was significantly improved by the biochar treatment, suggesting that the biochar treatment changes the relative abundance of *Pseudomonas*. The soil samples were separated into categories, which matched the biochar treatments (Fig. 4). Principle component analysis separated the biochar treatment and the control soils, suggesting that biochar treatment influences the bacterial community composition.

**Table 4:** Comparison (*t*-tests and Metastats) of different biochar treatments on genus abundance.

Phyla	Genus	Relative fold change	p value (*p < 0.05, **p < 0.01)
		B1/B0	
*Proteobacteria*	*Pseudomonas*	18.10	0.001**
	*Sphingomonas*	18.57	0.002**
	*Geobacter*	−2.75	0.007**
	*Nitrosospira*	−1.12	0.018*
	*Rhizobium*	1.24	0.033*
	*Rhodoplanes*	2.94	0.042*
*Gemmatimonadetes*	*Gemmatimonas*	2.54	0.005**
*Acidobacteria*	*Gp7*	2.03	0.030*
*Firmicutes*	*Pasteuria*	−12.17	0.021*
*Nitrospira*	*Nitrospira*	−10.54	0.033*
**Phyla**	**Genus**	**Relative fold change**	p value (*p < 0.05, **p < 0.01)
		**B2/B0**	
*Proteobacteria*	*Sphingomonas*	19.40	0.001**
	*Pseudomonas*	12.45	0.004**
	*Lysobacter*	2.67	0.004**
	*Geobacter*	−2.32	0.008**
	*Steroidobacter*	1.02	0.010**
	*Rhizobium*	1.83	0.033*
	*Nitrosospira*	−1.94	0.015*
	*Desulfocapsa*	−3.79	0.026*
	*Rhodoplanes*	1.66	0.039*
*Gemmatimonadetes*	*Gemmatimonas*	2.38	0.034*
*Acidobacteria*	*Gp7*	2.07	0.016*
*Firmicutes*	*Pasteuria*	−17.75	0.030*
*Nitrospira*	*Nitrospira*	−19.48	0.016*

## Discussion

Bacterial community composition and its relative proportion in soil microbial communities varied and was influenced by both the biochar treatment and number of continuous cropping years. This result is in accordance with the findings of previous studies^18, 19^ that also investigated the changes in the taxonomy of soil microbial communities after biochar amendment. Possible causes of the ecological shifts in the relative abundance of the bacterial community observed may include effects of the root exudates on a portion of the microbial community as a result of continuous cropping; changes to the physiochemical state of the soil environment, including pH, mineral content, pore and particle size; and changes to the water and nutrient availability due to the application of biochar and to the continuous cropping system, all of which have been shown to influence the composition of the bacterial community in soils^20^. Further work will be required to determine the relative importance of these factors in altering the composition of the microbial community due to biochar amendments to soils.

The relative abundance of *Acidobacteria* in the cotton soil continuously cropped for 14 years was approximately 57.3% to 184.3% higher compared with that of the 2-year continuously cropped soil (Fig. 2). Of the 15 significantly different genera found in the soils continuously cropped for 14 years and 2 years, 6 belong to *Acidobacteria* (Table 3). This result is probably due to the soil organic matter and especially the pH. The pH in cotton soil that has been continuously cropped for 14 years was significantly lower than that in the 2-year continuously cropped soil (Table S3). Soil pH has been recently documented in various soil samples as the major factor that determines soil bacterial diversity and composition. Previous studies^21–23^ reported that soil pH influences bacterial communities in soils across North and South America, in Britain and on Changbai Mountain. The effects of soil pH on the relative abundance of some bacterial groups in this study are consistent with these studies, which indicate that the relative abundance of *Acidobacteria* tends to increase with lower pH values^22–26^. Thus, our results further emphasize that soil pH plays an important role in shifting the composition of the bacterial community in the cotton soils with different continuous cropping years. However, Chan *et al*.^27^ reported that the addition of biochar to soil can significantly increase the pH of the bulk soil, which potentially provides a more favorable habitat for microbial organisms, especially bacteria that are sensitive to pH. This result is in accordance with the findings of our study. The application of biochar increased the pH of cotton soils that have been continuously cropped for a different number of years (Table S3).

Previous studies^3, 4, 28, 29^ have reported that high porosity, cation exchange capacity and sorption capacity of biochar provide a suitable habitat for microorganisms, promoting their activity in soil and affecting different microbial processes involved in nutrient cycling and organic matter decomposition. In this study, the four most abundant genera that were significantly affected by the biochar treatments were *Sphingomonas*, *Gemmatimonas*, *Nitrospira* and *Pseudomonas* (Table S2). Biochar treatment improved the relative abundance of *Sphingomonas* and *Pseudomonas* (Fig. 2), possibly due to biochar providing a suitable habitat. At the same time, the soil microbial communities are mostly limited by carbon sources, and biochar could provide abundant carbon resources for microbial growth; thus, the greater quantity of a few dominant microorganisms, such as *Sphingomonas* and *Pseudomonas*, as a result of the biochar treatment may also be due to improved carbon sources.

In addition, the bacterial genera *Sphingomonas* and *Pseudomonas* have been detected in a variety of environments. These genera are thought to be beneficial to plants, and they have been reported as potential antagonists of plant pathogens^30, 31^. Recently, these bacteria have been the focus of study due to their possible application in bioremediation^32^. Therefore, biochar application could possibly enhance cotton growth by improving bacterial genera abundance^1, 3^, which contributes to increased cotton productivity (Table S4). In addition, biochar is highly recalcitrant to microbial decomposition and thus guarantees a long-term benefit to soil fertility^33^. The actual effects of biochar application depend on various factors, such as the soil type and the water balance at a given site and possibly even the cultivated genotype, which currently require further studies.

## Material and Methods

### Site description

The experimental site was established at the experimental farm of the Industrial Crops Institute, Hubei Academy of Agricultural Sciences in Hubei Province, China (30°35′N, 114°37′E, 50 m a.s.l.). This region has a typical subtropical monsoon climate, with an average annual precipitation of 1269 mm and an average temperature between 15.8 °C and 17.5 °C.

### Soil and Biochar

**Figure 5:**
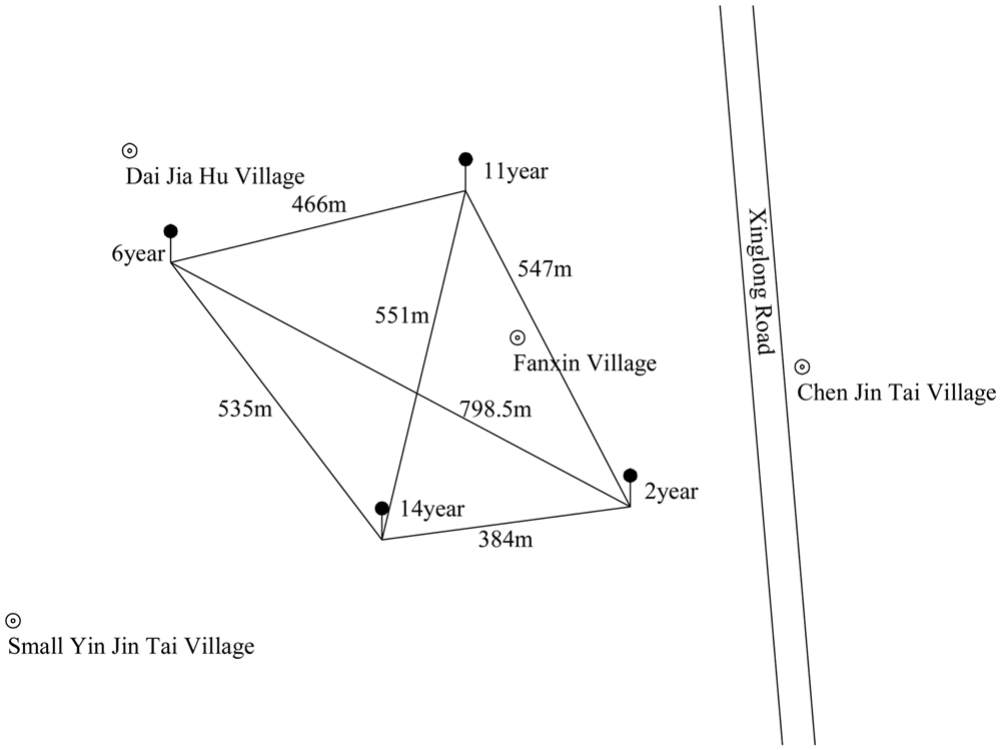
Location of sampling sites at the cotton research station in Qianjiang City (drawn by AutoCAD2004 software, the version number is 16.0).

The soil was collected from the surface layer of cotton soils (0–15 cm) that have been continuously cropped for 2, 6, 11 and 14 years at the cotton research station in Qianjiang City (Fig. 5). The collected soil was classified as acrisols according to the FAO, and the clay content was 66.3%. The original years of ‘2, 6 and 11-year soil’ was planted with corn, and the culturing and soil management were consistent. Corncob was used as the feedstock for biochar. The corncob was first air-dried and then pyrolyzed under controlled conditions to ensure uniform heating and treatment conditions. Biochar production was carried out using a traditional kiln reactor (Fengben Biological Technology Co., Ltd, Shandong, China) at a heating rate of 10 °C min^−1^ up to 550 °C. The basic properties of the collected soil and biochar are presented in Table 5.

**Table 5:** Basic chemical properties of biochar and soil.

Chemical Properties	Biochar	Contiuous cropping soil			
		2 Years	6 Years	11 Years	14 Years
Total N (g·kg^−1^)	2.7	1.31 ± 0.03^a^	0.93 ± 0.02^b^	0.61 ± 0.02^c^	0.63 ± 0.05^c^
NH_4_^+^ (mg·kg^−1^)	<0.1	15.2 ± 0.1^a^	13.1 ± 0.1^b^	11.2 ± 0.1^c^	10.8 ± 0.1^c^
NO_3_^−^ (mg·kg^−1^)	<0.2	2.3 ± 0.1^a^	2.3 ± 0.0^a^	1.8 ± 0.0^b^	1.70 ± 0.1^b^
Total C (g·kg^−1^)	680.0	10.5 ± 0.5^a^	9.2 ± 0.4^b^	9.1 ± 0.3^b^	9.0 ± 0.2^b^
pH (CaCl_2_)	8.3	6.3 ± 0.3^a^	6.1 ± 0.4^ab^	5.6 ± 0.1^b^	5.3 ± 0.1^b^
EC (μS·cm^−1^)	526.3	42.3 ± 2.7^a^	42.1 ± 1.6^a^	39.8 ± 2.0^a^	36.8 ± 3.0^b^

The data are expressed as mean ± SD (n = 3). Superscript letters that differ within rows indicate significant differences between treatments (p < 0.05).

### Pot experimental design

A pot trial was conducted at the experimental base of the Industrial Crops Institute, Hubei Academy of Agricultural Sciences in Hubei Province, China. This region has a typical subtropical monsoon climate, with an average annual precipitation of 1269 mm and average temperature between 15.8 °C and 17.5 °C.

After the removal of plant debris and stones, soils collected from different continuous cropping cotton fields (2, 6, 11 and 14 years) were mixed with biochar. The additive amount of collected soil in each experimental pot (the upper bore and pot height were 40.0 cm and 27.0 cm, respectively) was 7.5 kg of dry soil. The biochar application rates of each continuously cropped soil were 0 t·ha^−1^ (B0), 12.5 t·ha^−1^ (B1) and 20 t·ha^−1^ (B2), resulting in biochar weights of 0%, 1.538% and 3.077%, respectively, which accounted for the dry weights of the potted soil. In total, there were four continuous cropping soils, and each had three application rates of biochar; thus, there were twelve treatments in this experiment. Each treatment was replicated in ten pots; therefore, the twelve treatments employed 120 pots. Basal fertilizer was added to all pots. Equal amounts (10 g) of compound fertilizer (N:P:K = 15:15:15) were applied to all pots. The cotton seed was sown on 1 May 2014. One cotton (*Gossypium hirsutum* L.) seedling was planted per pot on 12 May 2014. The experimental pots were laid out in a randomized complete block design. Grasses and weeds were detached manually and left *in situ*, and water was added when necessary.

### Sample collection and preparation

The soil samplings were collected at the boll-opening stage (September 14th) in 2014 as follows: continuous cropping for 2 years with 0 t·ha^−1^ (2-B0), 12.5 t·ha^−1^ (2-B1) and 20 t·ha^−1^ (2-B2) biochar; continuous cropping for 6 years with 0 t·ha^−1^ (6-B0), 12.5 t·ha^−1^ (6-B1) and 20 t·ha^−1^ (6-B2) biochar; continuous cropping for 11 years with 0 t·ha^−1^ (11-B0), 12.5 t·ha^−1^ (11-B1) and 20 t·ha^−1^ (11-B2) biochar; and continuous cropping for 14 years with 0 t·ha^−1^ (14-B0), 12.5 t·ha^−1^ (14-B1) and 20 t·ha^−1^ (14-B2) biochar. For each treatment, soil samples (5 cm from the cotton trunk at a depth of 0–15 cm) were randomly collected from six of the ten replicates and mixed. Thus, a total of 12 mixed samples were obtained for the 12 treatments. The samples were immediately transported to the lab on ice and measured within one week after collection. Part of each soil sample was stored at −80 °C for soil microbiological analysis, and another part was air-dried, ground and passed through 1- and 2-mm mesh sieves for chemical analysis.

### DNA extraction and PCR amplification of 16 S rRNA

The genomic DNA was directly extracted from the soil using an E.Z.N.A.^®^ Soil DNA kit (Omega Bio-Tec, Inc., USA) according to the manufacturer’s instructions. The quality of the extracted DNA was preserved using 1% agarose gels. The V3–V4 hypervariable regions of 16 S rRNA were amplified via PCR from the microbial genomic DNA using barcoded fusion primers (forward primers:

341 F CCTACACGACGCTCTTCCGATCTN (barcode) CCTACGGGNGGCWGCAG, reverse primers: 805 R GACTGGAGTTCCTTGGCACCCGAGAATTCCAGACTACHVGGGTATCTAATCC). The reaction mixtures (50 µl) contained 5 µl of 10 × PCR reaction buffer (TakaRa, Japan), 10 ng of DNA template, 0.5 µl of each primer, 0.5 µl of dNTPs and 0.5 µl of Platinum Taq DNA polymerase (TakaRa, Japan). The PCR conditions were as follows: 94 °C for 3 min, 94 °C for 30 s, annealing at 45 °C for 20 s and 65 °C for 30 s, which was repeated for 5 cycles, followed by 94 °C for 20 s, 55 °C for 20 s and 72 °C for 30 s, which was repeated for 20 cycles, before a final elongation at 72 °C for 5 min. The PCR product was excised from the 1.5% agarose gel and purified using a QIAquick Gel Extraction Kit.

### Amplicon sequence and sequence data processing

The barcoded V3 and V4 amplicons were sequenced using the paired-end method with an Illumina MiSeq (Illumina, San Diego, CA, USA) system with a 6-cycle index. Sequences with an average Phred score of less than 25 that contain ambiguous bases, a homopolymer run exceeding 6, mismatches in primers or a length of less than 100 bp were removed using Prinseq software (PRINSEQ-lite 0.19.5). For the V3 and V4 paired-end reads, only the sequences that overlapped by more than 10 bp and without any mismatch were assembled according to their overlapping sequences using Flash software (FLASH v1.2.7). Reads that could not be assembled were discarded. Barcode and sequencing primers were trimmed from the assembled sequence (V3 and V4).

Sequences were clustered and assigned to operational taxonomic units (OTUs) at a 3% dissimilarity level using Uclust software (Uclust v1.1.579). Taxonomic ranks were assigned to each sequence using the Ribosomal Database Project (RDP) Naïve Bayesian Classifier v.2.2 trained on the Greengenes database (October 2012 version) (Lan *et al*. 2012). The relative abundance count at the genus level was log2-transformed and then normalized, as described in the following. The arithmetic mean of all transformed values was subtracted from each log-transform measured, and the difference was divided by the standard deviation of all log-transformed values for a given sample. After this procedure, the relative abundance profiles for all the samples exhibited a mean of 0 and a standard deviation of 1. Principal coordinates analysis (PCoA) at the genus level was performed using Bray-Curtis distances with Mothur 1.29.2 software.

### Soil characteristics

The total carbon and nitrogen were determined by combustion analysis (vario Macro CNS; Elementar, Germany). The ammonium (NH_4_^+^) and nitrate (NO_3_^−^) contents were determined through extraction with 0.5 M K_2_SO_4_ and colorimetrical analysis of NH_4_^+^ (Krom 1980; Searle 1984) and NO_3_^−^ (Kamphake *et al*. 1967; Kempers and Luft 1988) extracts using an automated flow injection Skalar Auto-analyzer (Skalar San Plus). The carbonate equivalence of the biochar was assessed using the method of Rayment and Lyons (2011). The electrical conductivity (EC) and pH of the biochar were determined in a 1:5 (w/v; g cm^−3^) soil:water environment and in 0.01 M CaCl_2_ mixtures, respectively.

### Statistical analysis

The results were analyzed using the SPSS software program (v10.0 for Windows, Chicago, IL, USA). The differences in the relative abundance of individual genera and the treatment means among plant age were tested using one-way variance analysis (ANOVA), and significant differences among the means were determined using the LSD test. Normal distribution and homogeneity of variance were verified by the Bartlett and Dunnett tests. The differences were considered statistically significant when p* < *0.05. T-tests and Metastats (http://metastats.cbcb.umd.edu/) in Mothur were used to compare the differences, and all p-values were adjusted with the false discovery rate (FDR) using the BH method with the mt.rawp2adjp function in R.

### Accession number of DNA sequence

The raw data has been submitted to a public repository (NCBI) and the accession number was SRP099813.

## Electronic supplementary material

Response of soil microbial community to application of biochar in cotton soils with different continuous cropping years
