# Supplementary material for: Response of soil microbial community to application of biochar in cotton soils with different continuous cropping years
Source: Sci Rep. 2017 Aug 31;7:10184. doi: 10.1038/s41598-017-10427-6 (PMC5578980; doi:10.1038/s41598-017-10427-6)
Supplement: Supplementary file 1 — Response of soil microbial community to application of biochar in cotton soils with different continuous cropping years [file 41598_2017_10427_MOESM1_ESM.doc]

**Response of soil microbial community to the application of biochar in the cotton soil with different continuous years**

Guangming Han*, Jiayang Lan*, Quanqiu Chen, Cui Yu, Shu Bie

Industrial Crops Institute of Hubei Academy of Agricultural Sciences, Wuhan 430064, China

*Correspondence to [mrhan888@hotmail.com](mailto:mrhan888@hotmail.com), 18986216860@163.com

Table S1 Raw reads, sequences and OTUs in cotton soils of continuous crop 2 years, 6 years, 11 years and 14 years treated with biochar (B0, 0 t·ha^-1^; B1, 12.5 t·ha^-1^; and B2, 20 t·ha^-1^).

| Group | Raw read | High quality sequence | OTUs |
| --- | --- | --- | --- |
| 2-B0 | 144321 | 138321 | 8791 |
| 2-B1 | 140968 | 136081 | 7833 |
| 2-B2 | 87323 | 83334 | 7127 |
| 6-B0 | 151799 | 145779 | 6832 |
| 6-B1 | 204627 | 196590 | 9420 |
| 6-B2 | 116775 | 113865 | 6552 |
| 11-B0 | 151243 | 144528 | 8850 |
| 11-B1 | 164973 | 159171 | 8914 |
| 11-B2 | 130443 | 125737 | 8196 |
| 14-B0 | 128535 | 125011 | 6991 |
| 14-B1 | 125661 | 121170 | 7443 |
| 14-B2 | 122647 | 118863 | 7016 |

Table S2 ANOVA for phyla abundance

| Genus | p value (***** p <0.05, ** p <0.01) | | |
| --- | --- | --- | --- |
|  | Continuous  cropping years | Biochar  treatments | Continuous cropping years * biochar treatments |
| Proteobacteria | 0.024***** | 0.019***** | 0.028***** |
| Acidobacteria | 0.016***** | 0.011***** | 0.011***** |
| Actinobacteria | 0.062 | 0.067 | 0.057 |
| Nitrospira | 0.024***** | 0.015***** | 0.018***** |
| Bacteroidetes | 0.034***** | 0.024***** | 0.028***** |
| Gemmatimonadetes | 0.064 | 0.068 | 0.058 |
| Planctomycetes | 0.002***** | 0.008***** | 0.001***** |
| Firmicutes | 0.013***** | 0.025***** | 0.019***** |
| Verrucomicrobia | 0.031***** | 0.021***** | 0.025***** |
| Chloroflexi | 0.072 | 0.057 | 0.063 |
| WS3 | 0.053 | 0.061 | 0.059 |
| Armatimonadetes | 0.059 | 0.068 | 0.057 |
| TM7 | 0.056 | 0.061 | 0.058 |

Table S3 The physicochemical properties in cotton soils of continuous crop 2 years, 6 years, 11 years and 14 years treated with biochar (B0, 0 t·ha^-1^; B1, 12.5 t·ha^-1^; and B2, 20 t·ha^-1^).

| Treatments | | B0 | B1 | B2 |
| --- | --- | --- | --- | --- |
| pH(CaCl_2_) | 2 years | 6.33±0.06b | 6.72±0.04a | 6.73±0.09a |
|  | 6 years | 6.13±0.01b | 6.70±0.08a | 6.81±0.02a |
|  | 11 years | 5.63±0.02b | 6.15±0.05a | 6.18±0.05a |
|  | 14 years | 5.36±0.07b | 5.93±0.09a | 6.09±0.04a |
| Organic matter (g kg^-1^) | 2 years | 15.36±0.51c | 18.42±0.62b | 21.64±0.11a |
|  | 6 years | 17.32±0.69c | 19.35±0.85b | 22.58±0.61a |
|  | 11 years | 14.29±0.29c | 16.33±0.78b | 18.82±0.51a |
|  | 14 years | 12.23±0.95c | 14.72±0.57b | 17.76±0.83a |
| Available N  (mg kg^-1^) | 2 years | 29.08±0.29b | 40.57±0.34a | 47.89±0.24a |
|  | 6 years | 31.12±0.98b | 39.01±0.12a | 41.24±0.10a |
|  | 11 years | 20.45±0.69c | 27.31±0.28b | 37.86±0.37a |
|  | 14 years | 14.56±0.69b | 26.38±0.79ab | 33.75±0.86a |
| Available P  (mg kg^-1^) | 2 years | 16.03±0.57b | 16.58±0.23b | 21.84±0.19a |
|  | 6 years | 16.46±0.59b | 17.96±0.47b | 19.35±0.43a |
|  | 11 years | 10.55±0.37c | 14.89±0.28b | 18.10±0.18a |
|  | 14 years | 9.21±0.10c | 12.25±.024b | 19.08±0.37a |
| Available K  (mg kg^-1^) | 2 years | 85.03±2.31b | 98.38±3.57ab | 109.25±1.97a |
|  | 6 years | 77.45±1.58b | 94.57±2.37ab | 100.13±3.24a |
|  | 11 years | 63.89±2.56c | 80.58±3.47b | 91.82±0.98a |
|  | 14 years | 60.05±4.01c | 83.88±1.29b | 94.35±2.05a |

The data are expressed as the mean ±SD (n=3). Superscript letters that differ within row indicate significant differences between treatments (p<0.05).

Table S4 Yield components and yield of cotton in cotton soils of continuous crop 2 years, 6 years, 11 years and 14 years treated with biochar (B0, 0 t·ha^-1^; B1, 12.5 t·ha^-1^; and B2, 20 t·ha^-1^).

| Treatments | | B0 | B1 | B2 |
| --- | --- | --- | --- | --- |
| Bolls per plant | 2 years | 6.92±0.21c | 8.23±0.15b | 9.63±0.14a |
|  | 6 years | 6.60±0.12c | 8.05±0.16b | 9.52±0.05a |
|  | 11 years | 6.43±0.10c | 7.62±0.04b | 9.15±0.11a |
|  | 14 years | 6.32±0.14c | 7.33±0.08b | 9.00±0.09a |
| Single boll weight /g | 2 years | 5.54±0.06b | 5.75±0.11ab | 5.83±0.08a |
|  | 6 years | 5.32±0.11b | 5.61±0.14ab | 5.71±0.08a |
|  | 11 years | 5.31±0.05b | 5.49±0.09a | 5.53±0.12a |
|  | 14 years | 5.21±0.06b | 5.40±0.02a | 5.44±0.03a |
| Lint percentage/% | 2 years | 39.92±1.23b | 40.15±1.56b | 42.36±2.01a |
|  | 6 years | 39.75±1.36b | 39.88±1.28b | 42.04±1.05a |
|  | 11 years | 38.14±1.67b | 39.49±2.36a | 39.82±2.03a |
|  | 14 years | 38.10±2.01b | 39.12±2.18a | 39.74±1.09a |
| Yield of per plant/g | 2 years | 40.33±1.59c | 42.74±1.28a | 43.13±2.03a |
|  | 6 years | 37.22±1.05b | 38.93±1.37b | 42.22±1.72a |
|  | 11 years | 35.70±2.06c | 37.15±1.98b | 40.15±1.78a |
|  | 14 years | 27.71±1.08c | 32.93±1.34b | 37.45±1.29a |

The data are expressed as the mean ±SD (n=3). Superscript letters that differ within row indicate significant differences between treatments (p<0.05).

**Figure S1.** Phyla relative abundance in cotton soils of continuous crop 2 years, 6 years, 11 years and 14 years treated with biochar (B0, 0 t·ha^-1^; B1, 12.5 t·ha^-1^; and B2, 20 t·ha^-1^).

**Figure S2.** Genus distribution in cotton soils of continuous crop 2 years, 6 years, 11 years and 14 years treated with biochar (B0, 0 t·ha^-1^; B1, 12.5 t·ha^-1^; and B2, 20 t·ha^-1^).
